# Diverse stoichiometry of dissolved trace metals in the Indian Ocean

**DOI:** 10.1038/srep01745

**Published:** 2013-04-29

**Authors:** Huong Thi Dieu Vu, Yoshiki Sohrin

**Affiliations:** 1 Institute for Chemical Research, Kyoto University, Uji, Kyoto 611-0011, Japan

## Abstract

Trace metals in seawater are essential to organisms and important as tracers of various processes in the ocean. However, we do not have a good understanding of the global distribution and cycling of trace metals, especially in the Indian Ocean. Here we report the first simultaneous, full-depth, and basin-scale section-distribution of dissolved (D) Al, Mn, Fe, Co, Ni, Cu, Zn, Cd, and Pb in the Indian Ocean. Our data reveal widespread co-limitation for phytoplankton production by DFe and occurrence of redox-related processes. The stoichiometry of the DM/phosphorus ratio agrees within a factor of 5 between deep waters in the Indian and Pacific, whereas it shows variability up to a factor of 300 among water masses within the Indian Ocean. This indicates that a consistent mechanism controls the stoichiometry in the deep waters, which are significantly depleted in Mn, Fe, and Co compared to requirements for phytoplankton.

Anumber of trace metals in seawater are essential to marine organisms for a variety of synergistic and antagonistic interactions^1^. Their concentrations have considerably varied throughout geological time, affecting biological evolution^2^. Trace metals in seawater are also important as tracers of a number of processes in the modern ocean, such as redox reaction, adsorption and scavenging, biological uptake and remineralization from organic matter, and submarine hydrothermal activity. Despite their importance, we do not have a good understanding of the global distribution and cycling of trace metals in the modern ocean^3^. This is particularly true in the Indian Ocean, which like the Pacific Ocean, is another region of upwelling in the global overturning circulation^4^. To study dissolved trace metals (DMs) in seawater, highly sensitive detection methods and the use of clean sampling and handling techniques are indispensable^5^. Reliable data of DMs in the Indian Ocean have been scarce, as follows: DAl^6^,^7^,^8^; DMn^9^,^10^,^11^; DFe^6^,^9^,^12^,^13^; DNi^10^,^14^; DCu^10^,^14^; DZn^10^,^14^,^15^; DCd^10^,^14^. There were no reliable data for DCo and DPb. The GEOTRACES program, an international study of marine biogeochemical cycles of trace metals and their isotopes (TEIs), has recently started to identify processes and quantify fluxes that control the distributions of key TEIs in the ocean, such as Al, Mn, Fe, Cu, Zn, Cd, and Pb, and to establish the sensitivity of these distributions to changing environmental conditions (http://www.geotraces.org/)^3^. The GEOTRACES JAPAN program conducted an ocean section study in the Indian Ocean during the KH-09-5 cruise of R/V Hakuho Maru from November 2009 to January 2010 in the northeast monsoon season. Recently Nishioka et al.^16^ have reported a basin-scale full-depth section profile of DFe that were measured onboard during this cruise by a FIA chemiluminescence detection system. Here we report the simultaneous, full-depth, and basin-scale section-distribution of dissolved Al, Mn, Fe, Co, Ni, Cu, Zn, Cd, and Pb that were measured on shore by a chelating resin extraction-ICP-MS method^17^.

## Results

### Distribution of DMs in the Indian Ocean

Clean seawater samples for trace metals were collected from station ER2 (86°E, 8.5°N) in the Bay of Bengal, station ER3 (80°E, 0°N) to the south of India, and stations ER5 (69°E, 14°N) to ER12 (58°E, 38°S) along a meridional transect from the Arabian Sea to the southern Indian Ocean (Supplementary Fig. 1). The samples include seawater from at least 7 major water masses in the Indian Ocean^4^ (Supplementary Fig. 2). Briefly, seawater samples were filtered through a filter with a pore size of 0.2 μm and acidified to pH 2.2 using hydrochloric acid immediately after sampling. Trace metals (Al, Mn, Fe, Co, Ni, Cu, Zn, Cd, and Pb) in seawater were simultaneously concentrated using a chelating resin^17^ and determined using a high resolution ICP-MS in our land laboratory. Ocean Data View (Schlitzer, R., http://odv.awi.de, 2012) was utilized for data analysis and preparation of some figures.

**Figure 1:**
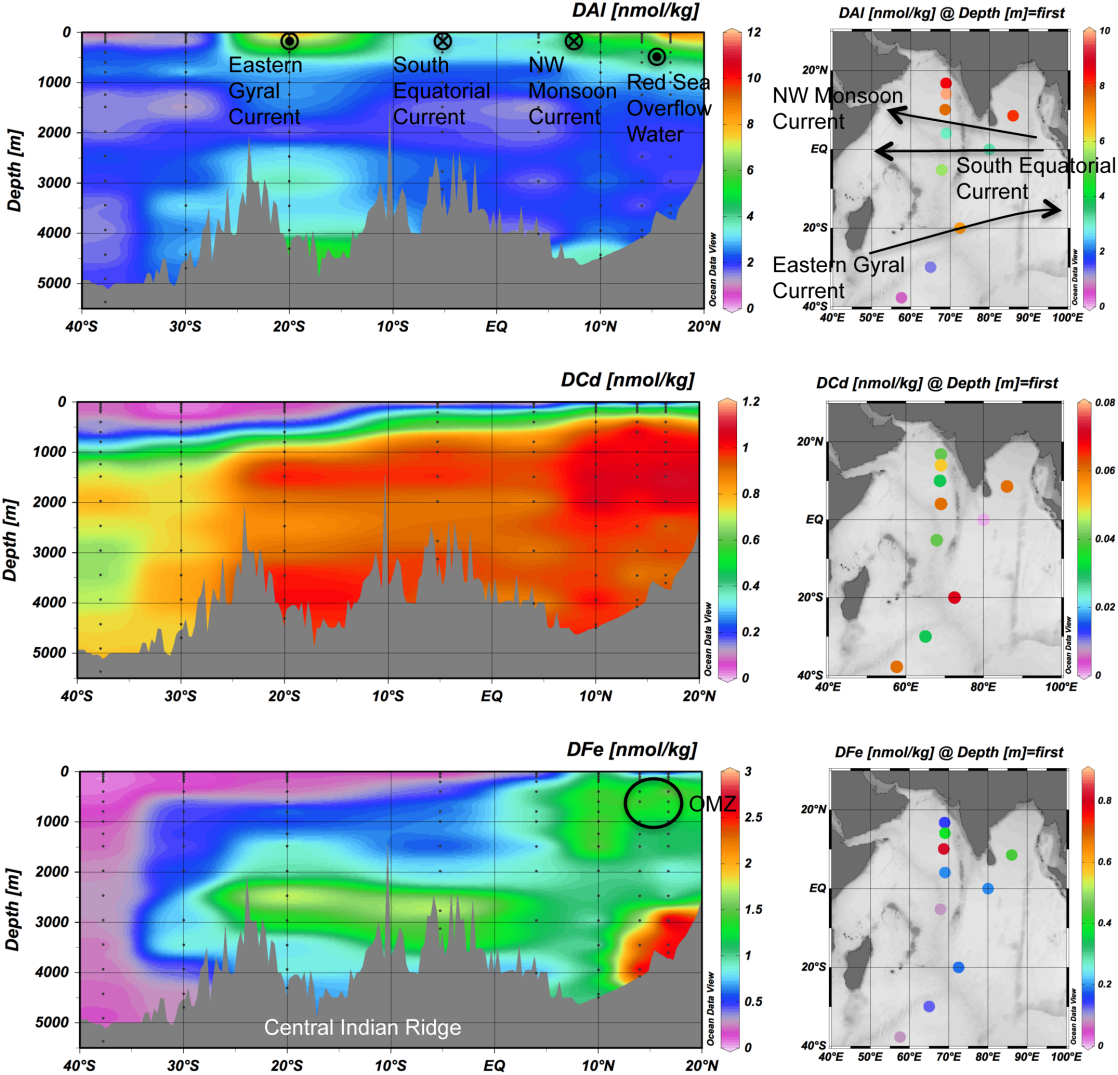
Meridional section (~70°E) and surface distributions of dissolved trace metals. Top, aluminium; middle, cadmium; bottom, iron. OMZ, oxygen minimum zone.

The 9 trace metals are divided into 3 groups based on distribution and correlation in concentration (Supplementary Fig. 3 and Supplementary Table 1). The meridional section (~70°E) and surface distributions of DAl, DCd, and DFe are shown in Fig. 1 as representative trace metals for each group (Supplementary Figs. 4 and 5 show all metals). (1) Group 1 includes scavenged-type trace metals Al, Mn, Co, and Pb. The concentrations of these metals are high in surface water, especially at northern stations, and relatively uniformly low in deep water. The vertical profiles of DAl and DMn are generally consistent with the abovementioned literature^6^,^7^,^8^,^9^,^10^,^11^. These 4 metals are supplied to surface water from the land and removed by scavenging. The correlation coefficients (*r*) are 0.67–0.73 for DAl, DMn, and DPb among each other (Supplementary Table 1). Only DCo exhibits a significant depletion in surface water, resulting in moderate correlations between DCo vs. the other metals (*r* = 0.39–0.67). It has been reported that Co plays a particularly important role in the growth of cyanobacteria, whereas the biochemical processes responsible for the major cellular utilization of Co in marine cyanobacteria are unknown^18^. The significant depletion of DCo in surface water indicates that the ratio of biological uptake to supply is higher for Co than for Al, Mn, and Pb. (2) Group 2 includes nutrient-type trace metals Ni, Cu, Zn, and Cd. These metals are characterized by high concentrations with a gradual northward increase in deep water. The DNi, DCu, and DZn show the strongest correlation with silicate (*r* = 0.81–0.97), and DCd shows the strongest correlation with phosphate (*r* = 0.97). The regression lines for DMs vs. major nutrients (P, N, and Si) agree very well with those reported in previous literature^10^,^14^ (Supplementary Table 2 and Supplementary Fig. 6). Biogeochemical cycling mainly controls the distribution of these metals: they are taken up by phytoplankton in surface water, transported downward by settling particles, remineralised in deep water, and due to these processes further increase in concentration with increasing age of the deep water. (3) Group 3 includes Fe, classified as a trace metal that is recycled and scavenged. The DFe is generally depleted in surface water and has great intra-basin variability, ranging from 0.03 to 2.87 nmol/kg. There is good linearity (*r* = 0.71, *n* = 132) between our DFe concentrations and those determined by chemical luminescence on board the vessel^16^ (Supplementary Fig. 8). The correlation coefficients for DFe are in the range from −0.28 to 0.33 with the scavenged-type metals and from 0.31 to 0.62 with major nutrients and the nutrient-type metals. The DFe shows a unique deep water maximum above the continental rise in the Arabian Sea (3000–4000 m depth, 12–20°N), which may be related to Fe sources at the continental margin characterized by a more reduced oxidation state^19^. Previously, Fe was assumed to be a nutrient-type trace metal and to have a constant concentration in deep water due to complex formation with organic ligands^20^. Our data demonstrate, however, that Fe has a unique distribution and is substantially affected by localized processes.

## Discussion

The dynamic range in surface DAl reaches 10 (Fig. 1). Surface maxima of DAl occur at northern stations and ~20°S, both extending to a depth of ~500 m, suggesting supply of Al from both aeolian dust and currents, such as the Northwest Monsoon Current and the Eastern Gyral Current^4^. The DMn, DCo, and DCu have similar surface distributions to DAl, suggesting the effect of coincident supply. The DPb has the highest surface concentrations between 10°N and 5°S unlike the other metals. This may be caused by high Pb load from anthropogenic sources. The DNi, DZn, and DCd are more uniformly distributed in surface water. Surface DFe reaches 0.8 nM at ER7 (69°E, 14°N), whereas it is less than 0.4 nmol/kg at other stations. The DFe/DAl ratio in surface water is 0.03–0.11, which is significantly lower than the Fe/Al ratio in the Arabian Sea aerosol (0.3)^21^. The surface depletion of DFe is attributed to uptake by phytoplankton. It is likely that supply of DFe to surface water was highest at the northernmost station ER5 as well as for DAl and other metals. However, active uptake of DFe by phytoplankton resulted in a low DFe concentration and a high biomass (up to 0.55 μg/kg in chlorophyll *a*) at this station. Over the study area the surface water is also depleted in silicate (< 1.0 μmol/kg) and dissolved inorganic nitrogen (sum of nitrate, nitrite, and ammonium < 0.6 μmol/kg). Takeda et al.^12^ conducted nutrient-enrichment bottle incubation at 65°E, 15°N (close to our station ER6) using ambient seawater that had a DFe concentration similar to our samples, and concluded that Fe and major nutrients are co-limiting phytoplankton production during the northeast monsoon. Taking account of their and our data, it is likely that Fe is a co-limiting factor for phytoplankton production over most of the study area.

**Figure 2:**
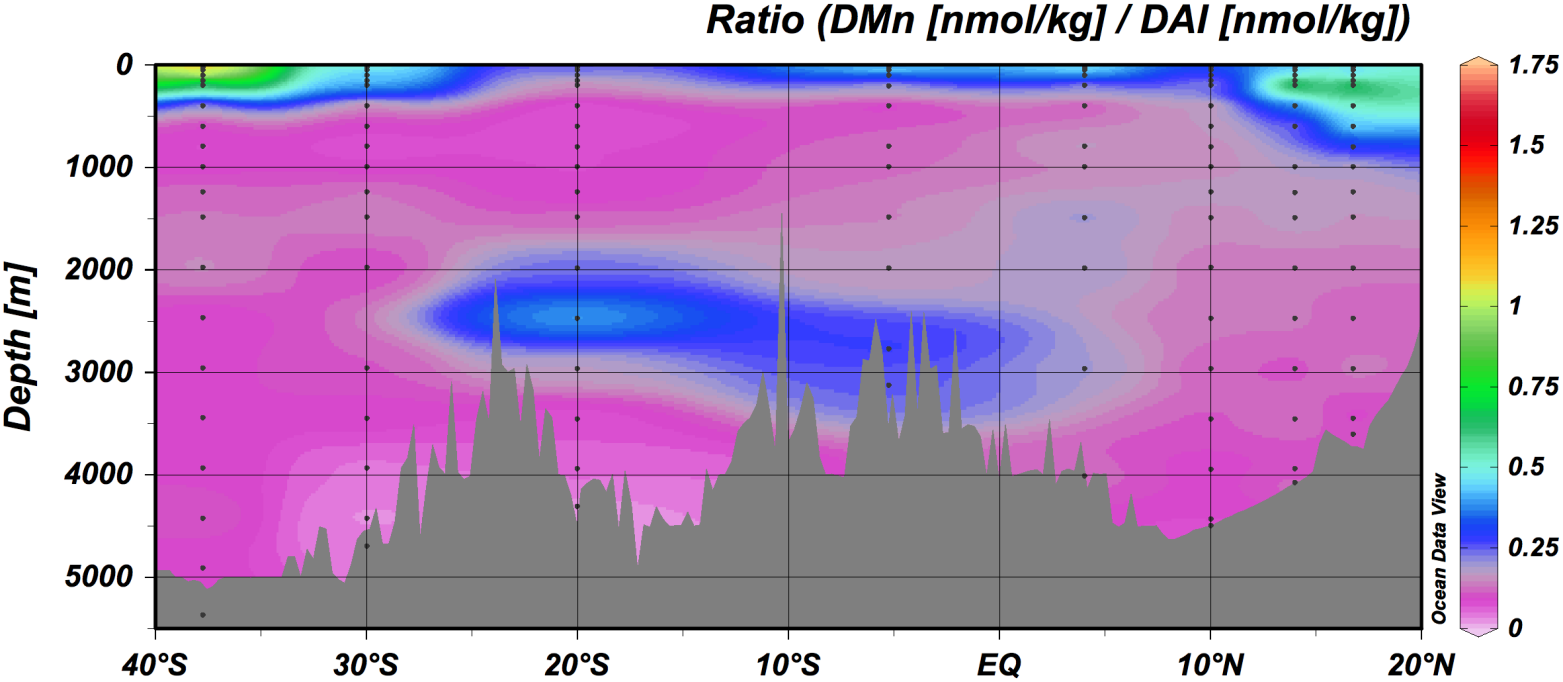
Meridional section distribution (~70°E) of the DMn/DAl ratio.

The effect of redox reactions is particularly apparent in the DMn/DAl ratio (Fig. 2). The ratio in surface water is 0.3–1.4, which is significantly higher than the Mn/Al ratio in the Arabian Sea aerosol (0.007)^21^, indicating high dissolution efficiency of dust-derived Mn due to photochemical reduction^22^. The oxygen minimum zone (OMZ) was observed at depths of 80–1500 m at stations in the Bay of Bengal and the Arabian Sea. Here the DMn and DCo show a sharp maximum at ~200 m depth together with nitrite. This is in perfect agreement with previous findings for Mn and nitrite^9^, and indicates manganese reduction by microorganisms is taking place as well as nitrate reduction at the top of OMZ, where particulate Mn is transformed into DMn (Supplementary Figs. 4, 7). While DFe shows a broad maximum in the OMZ, there are no significant correlations between the other metals and oxygen. Finally, DMn/DAl exhibits a deep maximum at ~2500 m depth above the Central Indian Ridge (10–20°S) corresponding with elevated DFe. The δ^3^He also increased in the same samples (Takahata unpublished data) and showed significant linear regression with DMn/DAl and DMn (*r* ~0.5, *p* < 0.01, *n* = 44), indicating effects of hydrothermal plumes. This observation is consistent with literature reporting hydrothermal activities in this region^16^,^23^,^24^,^25^,^26^,^27^. In the plume samples, a slight positive deviation is recognized for DAl probably due to resuspension of sediment material, since DAl/(DAl + DFe + DMn) vs. δ^3^He gives inverse linear regression (*r* ~0.7, *p* < 0.01, *n* = 44). Indistinct negative deviations are recognized for DNi, DZn, DCd, and DPb.

**Figure 3:**
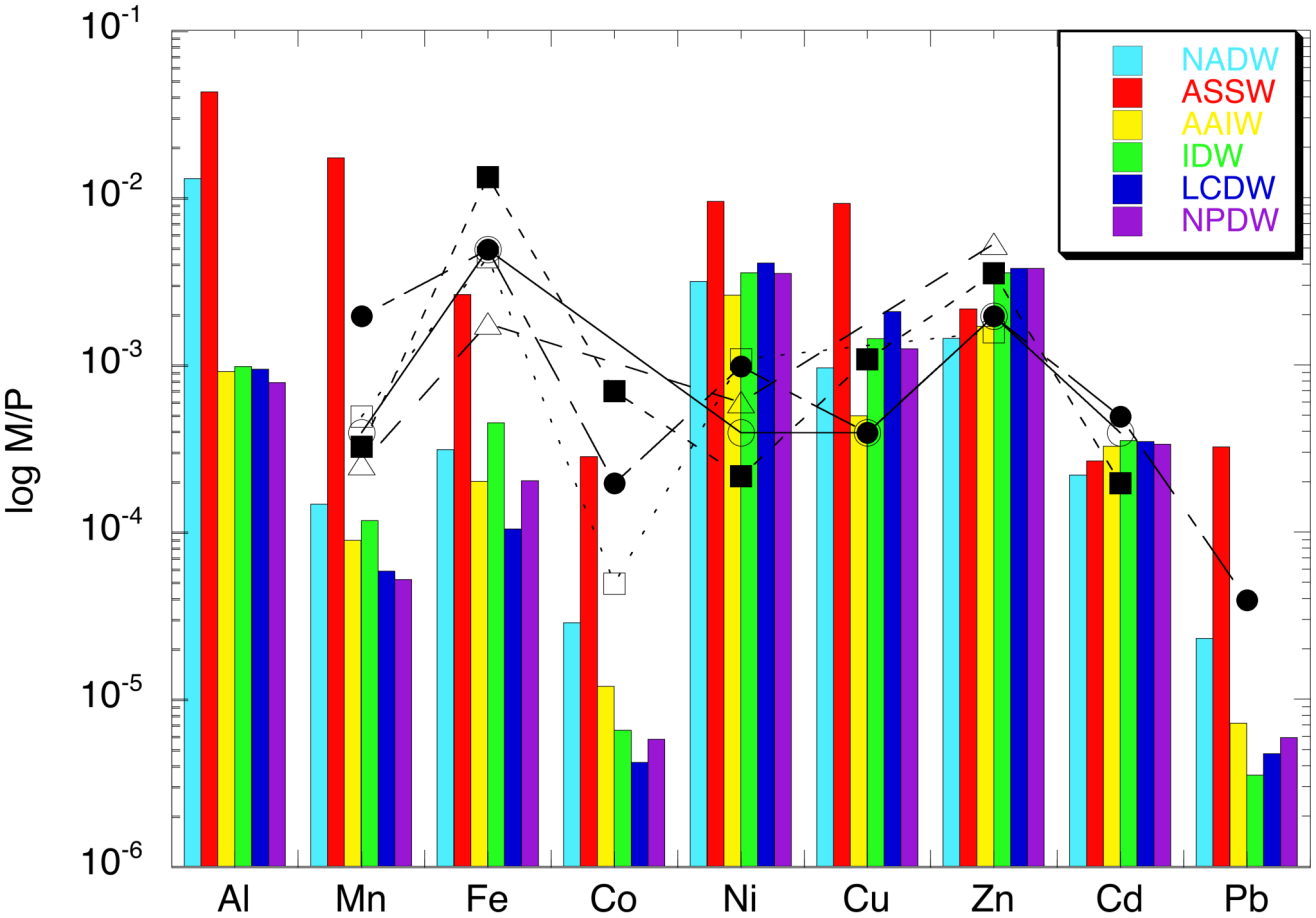
The M/P ratios of water masses and phytoplankton. Column plots for water masses: Arabian Sea Surface Water (ASSW), Antarctic Intermediate Water (AAIW), Indian Deep Water (IDW), and Lower Circumpolar Deep Water (LCDW) in the Indian Ocean, North Atlantic Deep Water (NADW), and North Pacific Deep Water (NPDW). The samples for NADW and NPDW were obtained from 64.05°W, 31.46°N and 170.00°W, 10.00°N, respectively, and analysed in our laboratory using the same method as this work. Scatter plots for phytoplankton: open circle, North Pacific^1^; closed circle, North Atlantic^29^; open triangle, Southern^30^; closed square, South China Sea^31^,^32^; open square, equatorial Pacific^33^.

Alfred C. Redfield found that the average molar ratio of C:N:P in organic matter of phytoplankton is close to 106:16:1, and that the dissolved ratio of N/P in seawater also generally follows this ratio^28^, termed the Redfield ratio. A basic question in chemical oceanography has been whether the Redfield ratio can be extended and consistent for trace metals as “an extended Redfield ratio”. The present data permit us to investigate the validity of extended Redfield ratio, using global stoichiometry data of DMs for the first time. The DM/P ratios for major water masses in the Indian Ocean show substantial intra-basin variation (Fig. 3). Surface waters, such as Arabian Sea Surface Water (ASSW), are characterized by high DM/P ratios; the DMn/P ratio exhibits the largest variation up to a factor of 300 between ASSW and Lower Circumpolar Deep Water (LCDW). North Atlantic Deep Water (NADP) that is freshly delivered by sinking within the global ocean circulation has relatively high DAl/P, DCo/P, and DPb/P ratios compared to the deep water masses, Indian Deep Water (IDW), LCDW, and North Pacific Deep water (NPDW), in the overturning Indian and Pacific Oceans. It should be noted that the DM/P ratios for all metals agree within a factor of 5 among IDW, LPDW, and remote NPDW. Fig. 3 also shows the M/P ratio that has been observed in organic matter of natural phytoplankton^1^,^29^,^30^,^31^,^32^,^33^. Recent studies proved that there are significant variations in metal quotas of phytoplankton depending on groups of phytoplankton, locations, and seasons^32^,^33^. However, we can see a generalized trend. The Cu/P, Zn/P, and Cd/P ratios are within the same order of magnitude for both phytoplankton and deep water, whereas the Mn/P, Fe/P, and Co/P ratios of phytoplankton can increase 100-fold or more compared to those in deep water. Usually upwelling deep water is a primary source of major nutrients for phytoplankton in euphotic zone. Our data suggest that sufficient amounts of Cu, Zn, and Cd would be supplied by deep water as well as major nutrients. However, Mn, Fe, and Co must be supplied from other sources when phytoplankton grow by utilizing major nutrients supplied by deep water, or they would be a limiting factor for the growth. In conclusion, although a simple extended Redfield ratio is unrealistic for trace metals, there is a systematic relationship. In follow up papers, we will determine total dissolvable and labile particulate concentrations of trace metals, discussing overall framework of the biogeochemical cycles of trace metals in the Indian Ocean.

## Methods

### Reagents and materials

Ultrahigh-purity HCl, HF, HNO_3_, HOAc, and NH_3_ (TAMAPURE, Tama Chemicals or Ultrapur, Kanto Chemical) were used for material cleaning, solution preparation, sample preservation, and analysis. Standard solutions of elements were prepared from commercially available standard solutions (1000 ppm; Wako Pure Chemical Industries). Deionized water (MQW) was purified with a Milli-Q gradient-A10 system (Millipore).

Experimental work was performed on a clean bench or in a clean room (class 1000) in order to avoid any contamination. Low-density polyethylene bottles (LDPE, Nalge Nunc) were used for storage of seawater and preparation of solutions. The bottles were cleaned successively with alkaline detergent and 4 M HCl. They were further cleaned successively with hot 2 M HF, hot 1 M HCl, hot 1 M HNO_3_ (all Ultrapure 100), and hot MQW using a microwave oven. A 30 ml LDPE bottle with a polypropylene cap made with methylaluminoxane catalysts was used to store the eluate containing extracted elements until determination. In order to minimise contamination of Al from the cap, cleaning the bottle with HF was conducted more carefully by turning the HF full-filled bottles upside down after the microwave heating.

Other materials, such as pipet tips, were cleaned in the same way. Teflon materials, such as PFA tubes, Teflon joints, and valves, were cleaned in a mixed acid (HF, H_2_SO_4_, and HNO_3_) at 200°C on a hot plate. Then, the materials were thoroughly rinsed with MQW, and further cleaned by the microwave cleaning steps listed above.

### Sample collection

Seawater samples were obtained during the GEOTRACES JAPAN cruise of R/V Hakuho Maru KH-09-5 from November 2009–January 2010. Seawater samples were collected with Niskin-X bottles mounted on a CTD-rosette water sampling system (General Oceanics), of which the frame was finished with epoxy paint^5^. The inside of the Niskin-X bottles was coated with Teflon and thoroughly cleaned with detergent and HCl at the beginning of the cruise. Temperature (T) was measured with the CTD sensor. Salinity (S) was determined by conductivity on board the vessel. Dissolved oxygen was determined by the Winkler method. Major nutrients were measured with an autoanalyzer. Chlorophyll *a* (Chl. *a*) was determined by fluorometry.

### Sample preparation and preconcentration procedure

Upon retrieval of the CTD-rosette water sampling system, the Niskin-X bottles were carried into a clean booth and seawater was transferred to 500 ml pre-cleaned LDPE bottles. The samples were immediately brought into a clean room (Class 1000) on the vessel. An aliquot of seawater (250 ml) was filtered through a 0.2 μm precleaned Nuclepore filter (Coaster) by N_2_ gas pressure using a closed filtration system, followed by acidification with HCl (TAMAPURE AA-10) to a final concentration of 0.01 M and pH 2.2. This subsample was used for the determination of DMs. The seawater samples were stored at room temperature in our laboratory until analysis.

Preconcentration of the trace metals was performed using a chelating resin on which ethylenediaminetriacetic and iminodiacetic acids are immobilised (NOBIAS CHELATE-PA1, Hitachi High-Technologies). The preconcentration method has been reported elsewhere^17^ and is briefly described below. Prior to preconcentration, 15 ml of 1 M HNO_3_ (TAMAPURE AA-10) and 30 ml of MQW were passed through the column. The pH in the column was further adjusted to 6.00 ± 0.05 by passing 40 ml of 0.05 M HOAc-NH_4_OAc buffer. Seawater samples were adjusted to pH 6.00 ± 0.05 just before the preconcentration. About 120 ml of the sample was loaded at a flow rate of 3 ml/min. Salts were washed out of the column with 40 ml of 0.05 M HOAc-NH_4_OAc buffer. Trace metals were then eluted with 15 ml of 1 M HNO_3_ (TAMAPURE AA-10). The eluate was stored in a 30 ml LDPE bottle and kept upright to prevent any aluminium contamination from the cap until HR-ICP-MS measurement.

### Instrumentation and quantitative determination

Instead of using an ICP quadrupole mass spectrometer as used in our original work^17^,^34^,^35^, a high-resolution ICP mass spectrometer equipped with a magnetic sector mass spectrometer (ELEMENT2, ThermoFisher) was used to determine the concentration of the trace metals in the eluate. The operating conditions and measurement parameters of the ELEMENT2 are shown in Supplementary Table 2. The isotopes used for the determination were ^27^Al, ^55^Mn, ^56^Fe, ^59^Co, ^60^Ni, ^63^Cu, ^66^Zn, ^114^Cd, and ^208^Pb. Other isotopes were also measured for cross checking, except mono-isotopic Al, Mn, and Co. A medium resolution mode was employed to determine ^27^Al, ^55^Mn, ^56^Fe, ^59^Co, ^60^Ni, ^63^Cu, ^66^Zn, and ^114^Cd, while a low-resolution mode was used for ^208^Pb. The settings allowed for the resolution of isobaric interferences such as ^40^Ar^16^O and ^40^Ca^16^O for ^56^Fe, or ^98^Mo^16^O for ^114^Cd. Calibration lines for trace metals were obtained using standard solutions containing 1 M HNO_3_ (TAMAPURE AA-10). The standard solutions were measured at appropriate intervals during the analysis sequence to correct changes in background and sensitivity.

### Procedure blank, recovery and analysis of reference materials

The procedure blank was determined using MQW as a sample. The procedure blanks and detection limits are shown in Supplementary Table 3. The detection limit for HR-ICP-MS without preconcentration was evaluated to be three times the standard deviation for 1 M HNO_3_ (TAMAPURE AA-10). For Ni and Cd, the blanks in the eluate were less than the detection limits, whereas significant blanks were observed for Al, Fe, Mn, Co, Cu, Zn, and Pb. The overall detection limit is equal to the higher value for detection limit by HR-ICP-MS and by procedural blank. Compared to the observed concentration ranges in the samples, the blanks and overall detection limits were sufficiently low that the analytical results were not corrected for the blanks.

MQW and filtered seawater spiked with the target metals were used for recovery experiments. The targeted metals were recovered at 100 ± 1% from MQW (*n* = 3) and 100 ± 6% from seawater (*n* = 3). The analytical results of SAFe intercalibration seawater samples^36^ are shown in Supplementary Table 4. The results for the SAFe samples agreed well with the consensus values (http: www.geotraces.org/science/intercalibration/322-standards-and-reference-materials).

## Author Contributions

H.T.D.V. carried out sampling, determination of trace metals, and data analysis. Y.S. managed the study and wrote the manuscript.

## Supplementary Material

Supplementary Information Supplementary Information
