# Supplementary material for: Diverse stoichiometry of dissolved trace metals in the Indian Ocean
Source: Sci Rep. 2013 Apr 29;3:1745. doi: 10.1038/srep01745 (PMC3638160; doi:10.1038/srep01745)
Supplement: Supplementary Information [file srep01745-s1.pdf]

Supplementary Information

Diverse stoichiometry of dissolved trace metals in the Indian Ocean

Huong Thi Dieu Vu and Yoshiki Sohrin*

Institute for Chemical Research, Kyoto University, Uji, Kyoto 611-0011, Japan. Correspondence and requests for materials should be addressed to Y.S. (e-mail: sohrin@scl.kyoto-u.ac.jp).

Supplementary Table 1

The correlation matrix of dissolved trace metals and oceanographic parameters for all data in the Indian Ocean (n = 163).

	Depth	CTD Fluorescence	CTD Turbidity	Salinity	Potential Temperature	Potential Density Anomaly	O2	pH	Si(OH)4	NO3	NO2	NH4	DIN	PO4	DAl	DMn	DFe	DCo	DNi	DCu	DZn	DCd	DPb
Depth	1.00																						
CTDFluorescence	-0.41	1.00																					
CTDTurbidity	-0.04	-0.03	1.00																				
Salinity	-0.61	0.45	0.08	1.00																			
Potential Temperature	-0.79	0.57	0.03	0.77	1.00																		
Potential Density Anomaly	0.66	-0.57	-0.01	-0.65	-0.96	1.00																	
O2	0.18	0.17	-0.26	-0.03	0.11	-0.23	1.00																
pH	-0.21	0.27	0.32	0.23	0.23	-0.17	-0.01	1.00															
Si(OH)4	0.91	-0.45	0.02	-0.63	-0.86	0.76	-0.09	-0.24	1.00														
NO3	0.61	-0.56	0.09	-0.66	-0.86	0.85	-0.52	-0.24	0.78	1.00													
NO2	-0.13	0.04	0.14	0.20	0.12	-0.06	-0.26	0.08	-0.12	-0.14	1.00												
NH4	0.03	0.08	-0.09	0.00	0.02	-0.04	0.14	-0.08	-0.03	-0.10	-0.02	1.00											
DIN	0.60	-0.56	0.10	-0.65	-0.86	0.85	-0.55	-0.24	0.78	1.00	-0.04	-0.10	1.00										
PO4	0.56	-0.52	0.15	-0.57	-0.81	0.82	-0.63	-0.21	0.76	0.97	0.06	-0.10	0.98	1.00									
DAl	-0.36	0.29	0.14	0.52	0.61	-0.62	-0.08	0.07	-0.41	-0.42	0.15	-0.03	-0.41	-0.37	1.00								
DMn	-0.45	0.44	0.13	0.65	0.67	-0.65	-0.06	0.12	-0.47	-0.57	0.53	0.02	-0.53	-0.43	0.73	1.00							
DFe	0.23	-0.26	0.17	-0.12	-0.33	0.36	-0.60	-0.05	0.43	0.51	0.16	-0.03	0.53	0.60	0.01	-0.01	1.00						
DCo	-0.42	0.17	0.19	0.42	0.35	-0.25	-0.61	0.08	-0.32	-0.06	0.56	-0.05	-0.01	0.09	0.53	0.67	0.33	1.00					
DNi	0.84	-0.51	0.08	-0.67	-0.88	0.80	-0.21	-0.23	0.96	0.86	-0.11	-0.08	0.85	0.83	-0.37	-0.49	0.49	-0.25	1.00				
DCu	0.90	-0.23	0.03	-0.42	-0.56	0.41	0.14	-0.15	0.81	0.46	-0.07	-0.01	0.46	0.43	-0.07	-0.18	0.31	-0.23	0.76	1.00			
DZn	0.94	-0.43	0.01	-0.66	-0.85	0.72	0.02	-0.23	0.97	0.73	-0.14	-0.01	0.73	0.69	-0.40	-0.48	0.38	-0.37	0.94	0.86	1.00		
DCd	0.60	-0.50	0.17	-0.57	-0.82	0.81	-0.58	-0.18	0.80	0.94	0.04	-0.05	0.95	0.97	-0.34	-0.40	0.62	0.06	0.88	0.49	0.74	1.00	
DPb	-0.69	0.57	0.02	0.70	0.94	-0.94	0.12	0.18	-0.78	-0.80	0.12	0.12	-0.79	-0.75	0.67	0.70	-0.28	0.39	-0.81	-0.45	-0.76	-0.75	1.00

Supplementary Table 2

The linear relation between dissolved trace metals and major nutrients in the Indian Ocean.

y = ax + b								
y	x	a	b	r	n	Locations	Depth range (m)	Authors
Ni (nM)	Si (μM)	0.054	2.47	0.93	102	all stations (14°30'N-23°30'N, 59°00'E-67°00'E)	0-4000	Saager et al. 1992
Ni (nmol/kg)	Si (μmol/kg)	0.054	2.44	0.96	167	all stations	10-5365	This study
Cu (nM)	Si (μM)	0.019	-0.13	0.95	9	22°30'N, 60°40'E	250-3000	Saager et al. 1992
Cu (nM)	Si (μM)	0.015	0.74	0.94	137	all stations (6°09'S-27°00'S, 50°54'E-56°58'E)	6-4975	Morley et al. 1993
Cu (nmol/kg)	Si (μmol/kg)	0.020	2.44	0.81	167	all stations	10-5365	This study
Zn (nM)	Si (μM)	0.06	0.50	0.89	50	all stations (14°30'N-23°30'N, 59°00'E-67°00'E)	0-4000	Saager et al. 1992
Zn (nM)	Si (μM)	0.049	1.3	0.88	131	all stations (6°09'S-27°00'S, 50°54'E-56°58'E)	6-4975	Morley et al. 1993
Zn (nM)	Si (μM)	0.0592	0.0028	0.96		all stations (~30°S, 30°E-115°E)	0-1000	Gosnell et al. 2012
Zn (nmol/kg)	Si (μmol/kg)	0.064	0.42	0.97	167	all stations	10-5365	This study
Cd (nM)	P (μM)	0.50	-0.49	0.97	14	14°30'N, 67°00'E	100-4000	Saager et al. 1992
Cd (nM)	P (μM)	0.15	-0.05	1.00	4	14°30'N, 67°00'E	0-100	Saager et al. 1992
Cd (nM)	P (μM)	0.50	-0.46	0.97	10	21°16'N, 63°22'E	30-3360	Saager et al. 1992
Cd (nM)	P (μM)	0.87	-1.26	0.96	10	22°30'N, 60°40'E	50-3000	Saager et al. 1992
Cd (nM)	P (μM)	0.16	-0.01	0.97	5	22°30'N, 60°40'E	0-50	Saager et al. 1992
Cd (nM)	P (μM)	0.28	-0.028	0.96	135	all stations (6°09'S-27°00'S, 50°54'E-56°58'E)	6-4975	Morley et al. 1993
Cd (nmol/kg)	P (μmol/kg)	0.35	-0.03	0.97	166	all stations	10-5365	This study

Supplementary Table 3
HR-ICP-MS instrumentation and operating conditions.

RF power	1200 W
Sampler cone	Ni
Skimmer cone	Ni
Nebuliser	PFA MicroFlow PFA-100
Sample take up rate	100 µl/min
Spray chamber	PFA Teflon
Plasma gas flow	16 l/min
Auxiliary gas flow	0.8 l/min
Nebuliser gas flow rate	Daily optimized for ^{7}Li, ^{115}In, and ^{238}U
Touch position	Daily optimized for ^{7}Li, ^{115}In, and ^{238}U
Lenses	Daily optimized for ^{7}Li, ^{115}In, and ^{238}U
Isotopes	
^{27}Al	Medium resolution, R = 4000
^{55}Mn	Medium resolution, R = 4000
^{56}Fe, ^{57}Fe	Medium resolution, R = 4000
^{59}Co	Medium resolution, R = 4000
^{60}Ni, ^{62}Ni	Medium resolution, R = 4000
^{63}Cu, ^{65}Cu	Medium resolution, R = 4000
^{66}Ni, ^{68}Ni	Medium resolution, R = 4000
^{111}Cd, ^{114}Cd	Medium resolution, R = 4000
^{206}Pb, ^{208}Pb	Low resolution, R = 600
Search window	150 % (Low resolution), 50 % (Medium resolution)
Integration window	60 % (Low resolution), 60 % (Medium resolution)
Take up time	60 s
Sample time	0.01 s
Sample per peak	10 (Low resolution), 20 (Medium resolution)
Pass	3
Run	3
Scan (Pass × Run)	3 × 3 = 9

Supplementary Table 4
Procedure blank and detection limit for 8-fold preconcentration.

Element	Procedure blank[a]	Detection limit		
		by HR-ICP-MS[b]	by procedure blank[c]	Overall
Al (nmol/kg)	0.19 ± 0.01	0.18	0.02	0.18
Mn (nmol/kg)	nd[e]	0.002	nd	0.002
Fe (nmol/kg)	nd	0.07	nd	0.07
Co (pmol/kg)	nd	2.2	nd	2.2
Ni (nmol/kg)	nd	0.16	nd	0.16
Cu (nmol/kg)	nd	0.01	nd	0.01
Zn (nmol/kg)	0.02 ± 0.01	0.02	0.04	0.04
Cd (nmol/kg)	nd	0.03	nd	0.03
Pb (pmol/kg)	0.2 ± 0.01	0.2	0.03	0.2

[a]mean ± SD (n = 3)
[b]8 times the 3SD for 1M HNO_3 (n = 20)
[c]3SD for the procedure blank
[d]not detected

Supplementary Table 5
Results on analysis of SAFe intercalibration seawater samples.

Element	Surface water S167		Deep water D173	
	This study[a]	Concensus value	This study[a]	Concensus value
Al (nmol/kg)	1.86 ± 0.05	1.74 ± 0.09	1.14 ± 0.10	1.04 ± 0.10
Mn (nmol/kg)	0.87 ± 0.04	0.83 ± 0.08	0.39 ± 0.01	0.37 ± 0.07
Fe (nmol/kg)	0.11 ± 0.00	0.09 ± 0.01	0.88 ± 0.10	0.92 ± 0.03
Co (pmol/kg)	4 ± 0	4 ± 2	45 ± 2	43 ± 3
Ni (nmol/kg)	2.30 ± 0.15	2.39 ± 0.09	9.14 ± 0.08	8.77 ± 0.31
Cu (nmol/kg)	0.56 ± 0.01	0.55 ± 0.04	2.37 ± 0.07	2.35 ± 0.19
Zn (nmol/kg)	0.08 ± 0.00	0.07 ± 0.01	7.23 ± 0.02	7.19 ± 0.70
Cd (pmol/kg)	1 ± 0	1	1007 ± 42	992 ± 35
Pb (pmol/kg)	49 ± 4	47 ± 3	28 ± 1	28 ± 2

[a]mean ± SD (n = 3)

Supplementary Figures and Legends

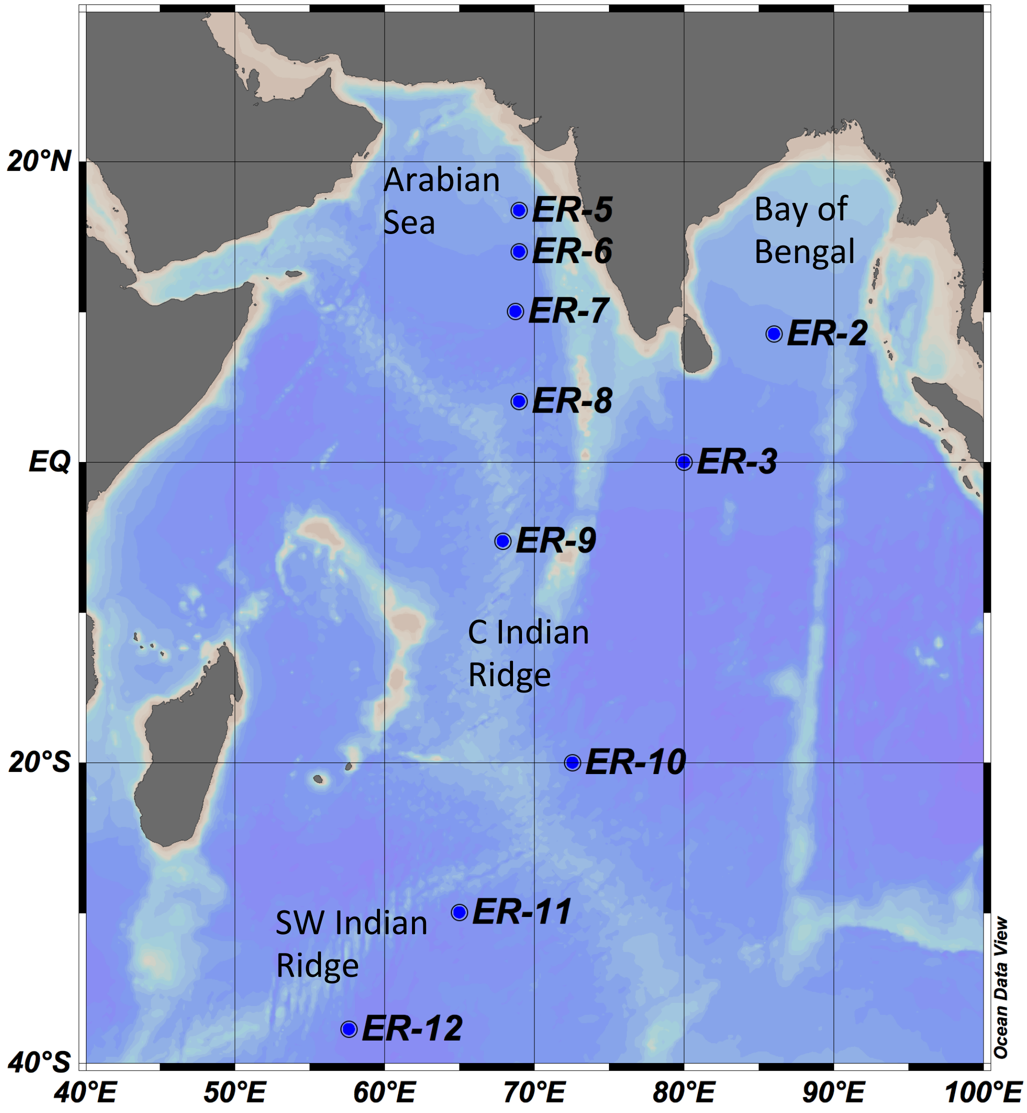

Supplementary Figure 1. Sampling locations in the Indian Ocean during the KH-09-5 cruise of R/V Hakuho Maru.

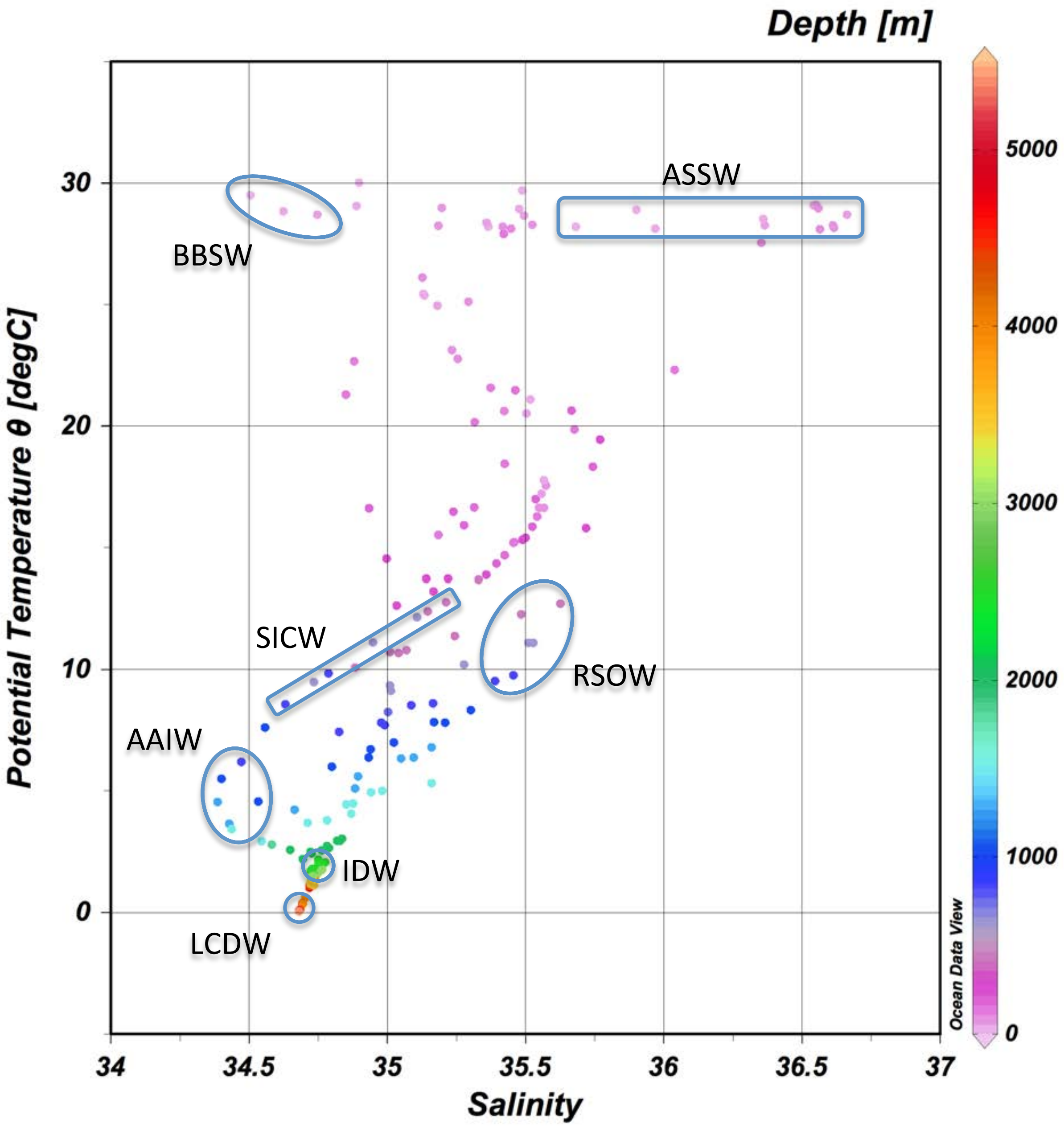

Supplementary Figure 2. T-S diagram for the samples collected from the Indian Ocean and major water masses. ASSW, Arabian Sea surface water; BBSW, Bay of Bengal surface water; RSOW, Red Sea Overflow Water; SICW, South Indian Central Water; AAIW, Antarctic Intermediate Water; IDW, Indian Ocean Deep Water; LCDW, Lower Circumpolar Deep Water.

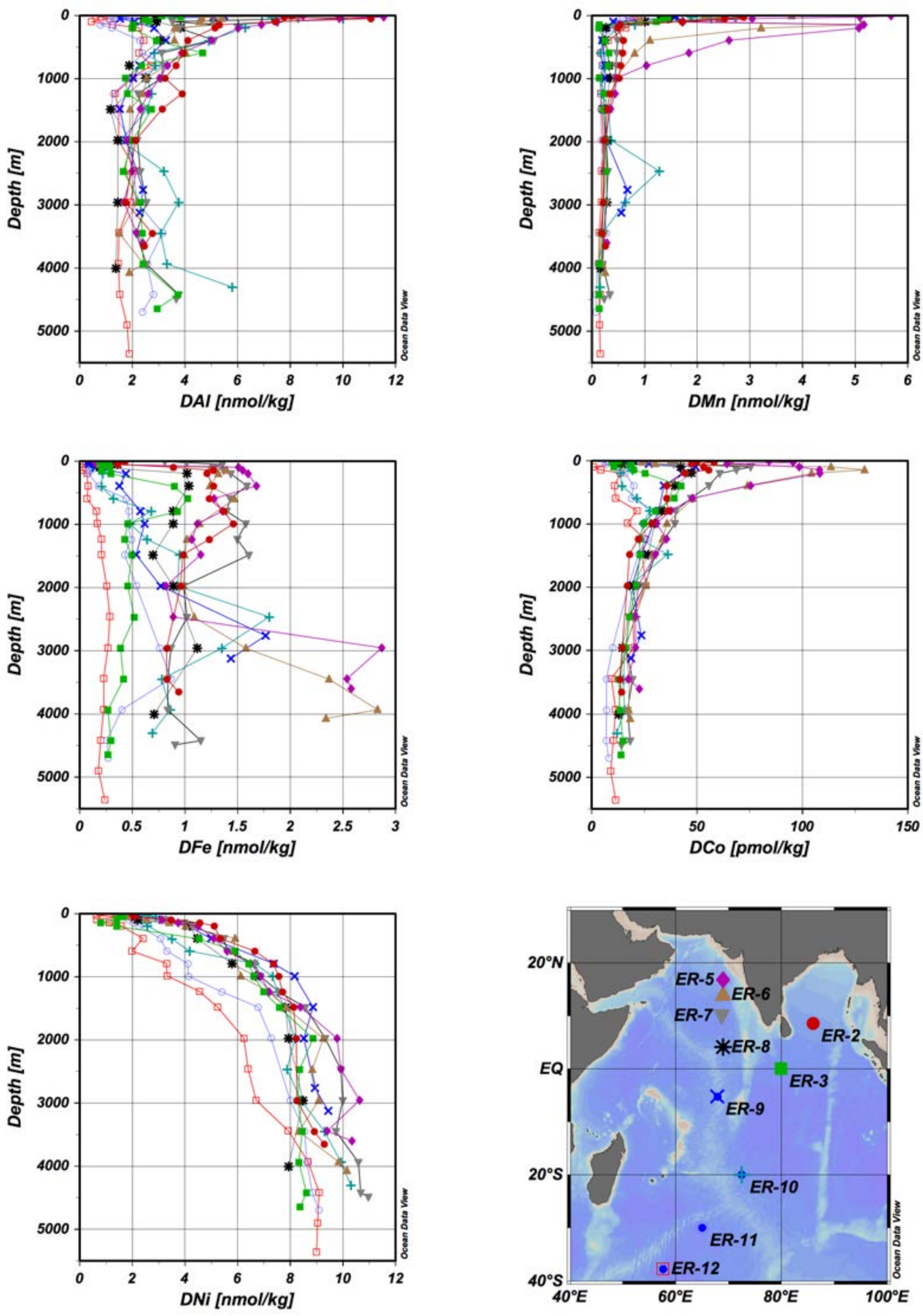

Supplementary Figure 3 a. Vertical profiles of DAl, DMn, DFe, DCo, and DNi at each station in this study.

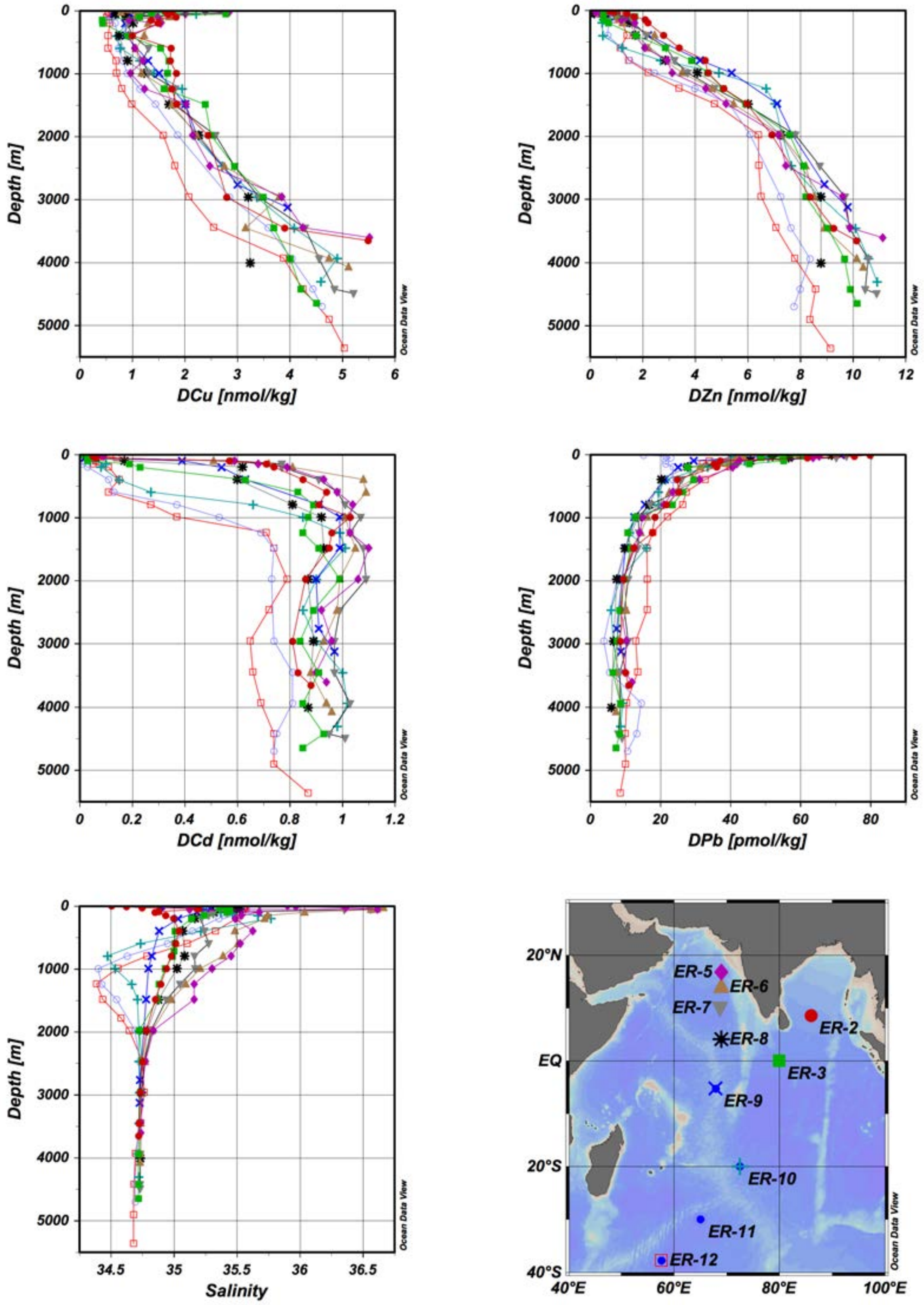

Supplementary Figure 3 b. Vertical profiles of DCu, DZn, DCd, DPb, and salinity at each station in this study.

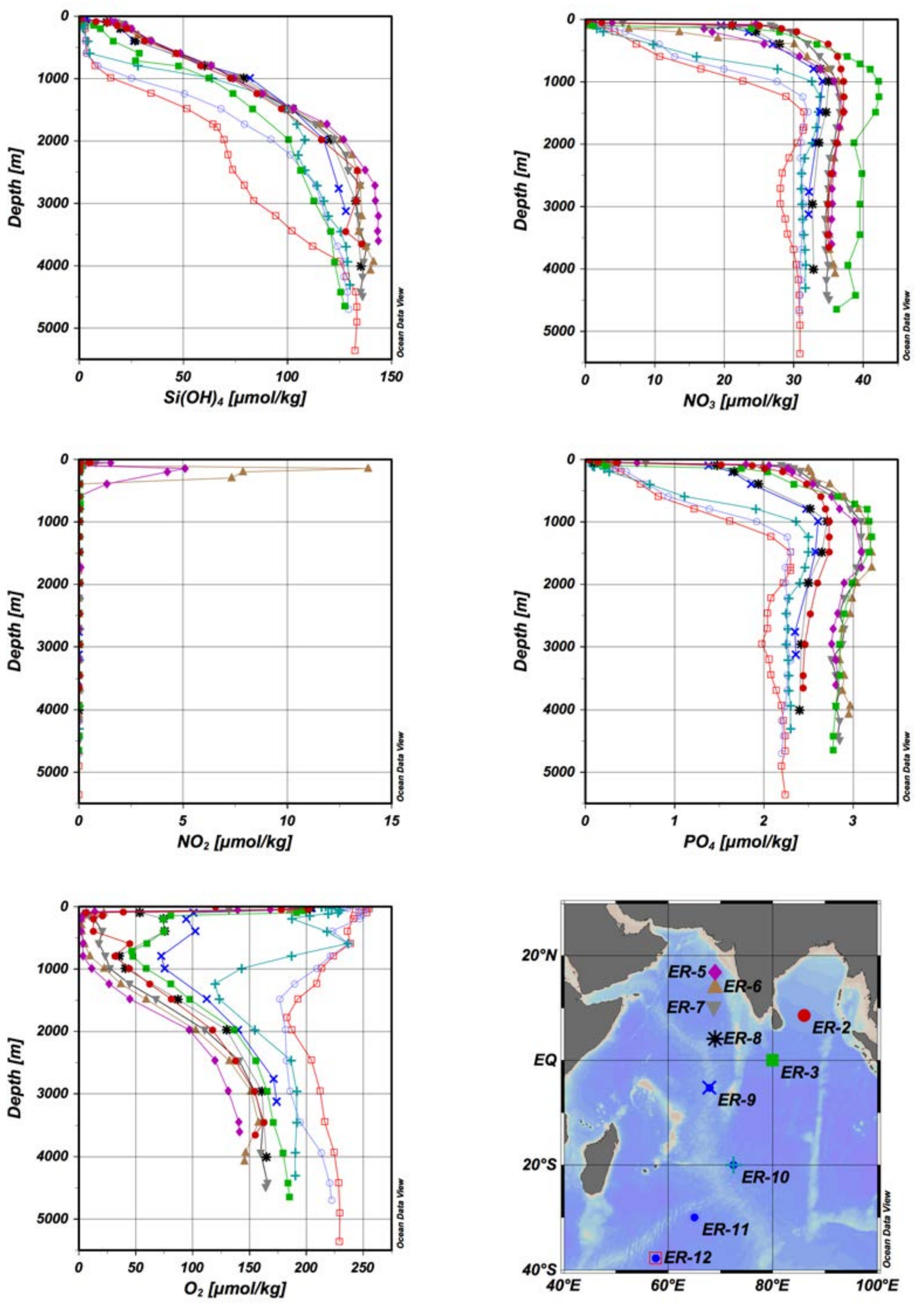

Supplementary Figure 3 c. Vertical profiles of silicate, nitrate, nitrite, phosphate and oxygen at each station in this study.

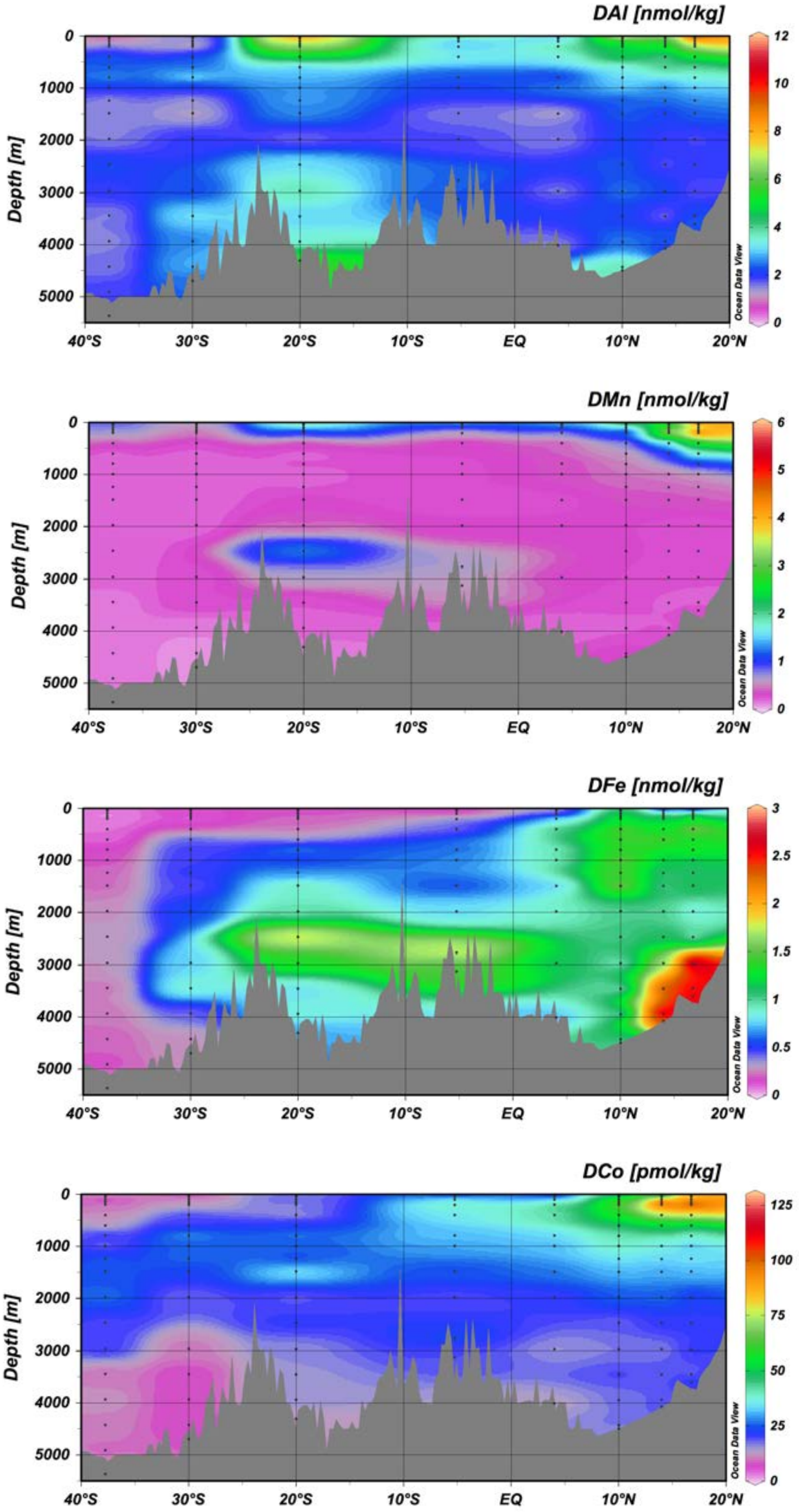

Supplementary Figure 4 a. Meridional section (~70°E) distribution of DAl, DMn, DFe, and DCo.

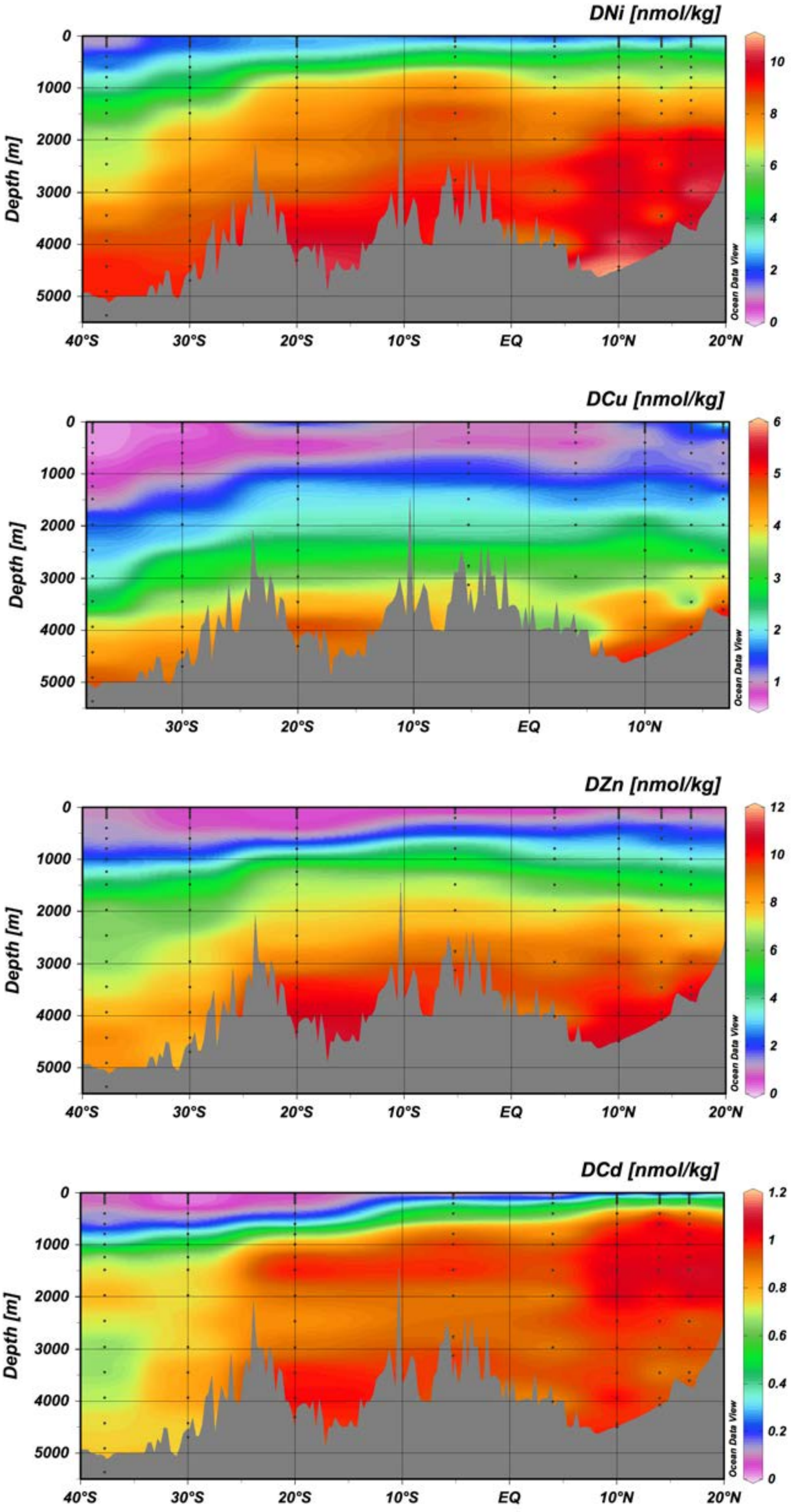

Supplementary Figure 4 b. Meridional section (~70°E) distribution of DNi, DCu, DZn, and DCd.

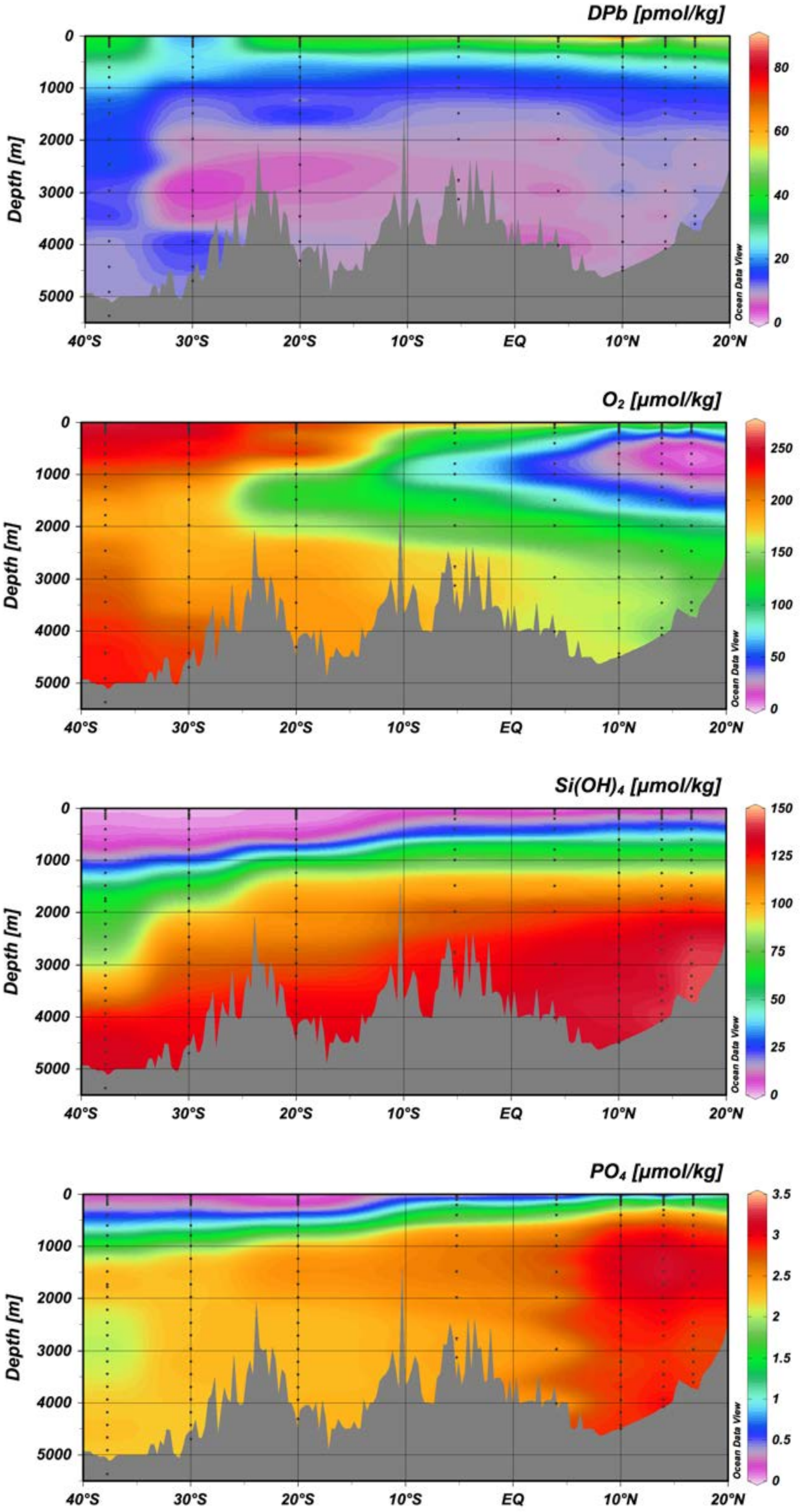

Supplementary Figure 4 c. Meridional section (~70°E) distribution of DPb, oxygen, silicate, and phosphate.

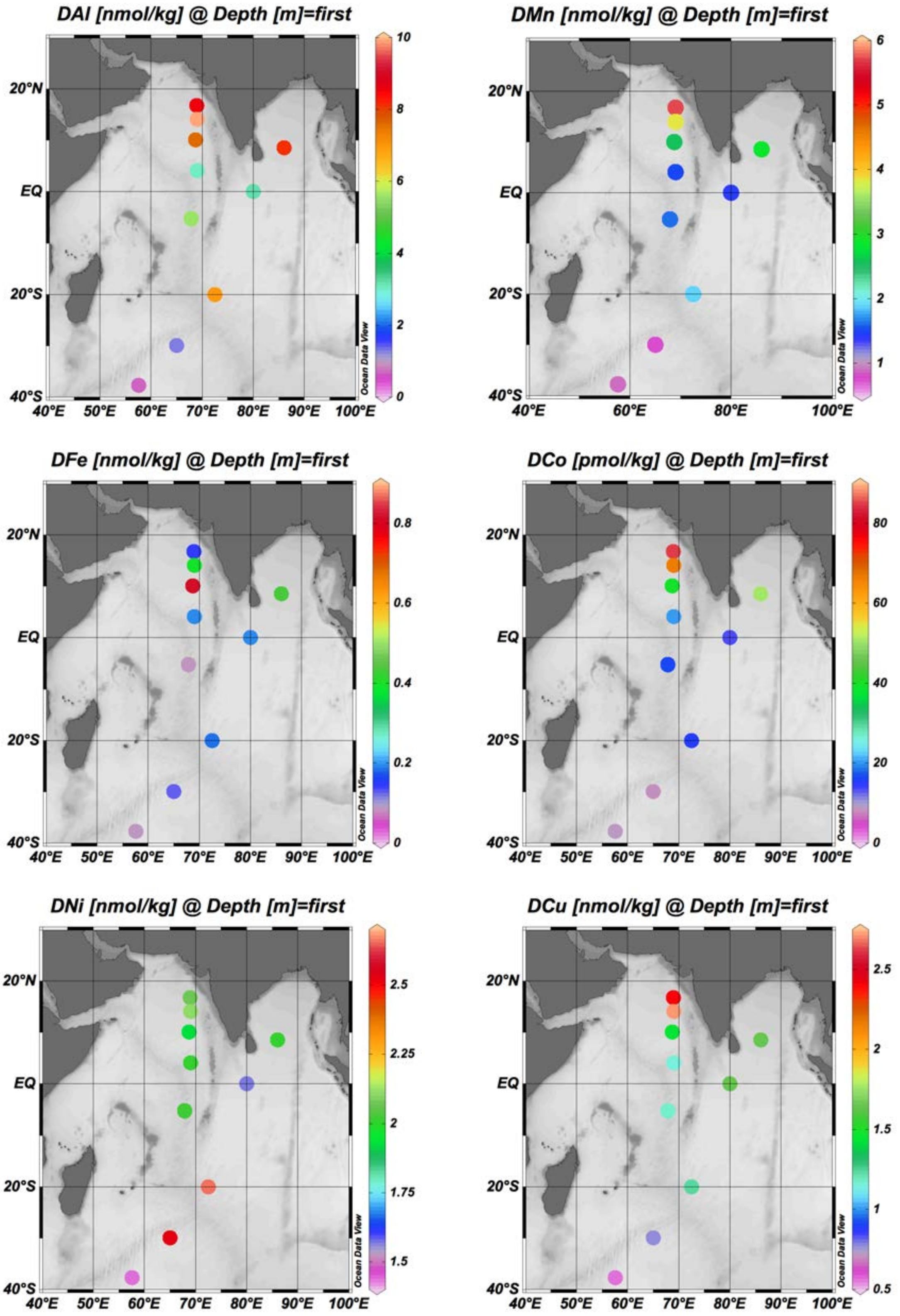

Supplementary Figure 5 a. Surface distribution of DAl, DMn, DFe, DCo, DNi, and DCu.

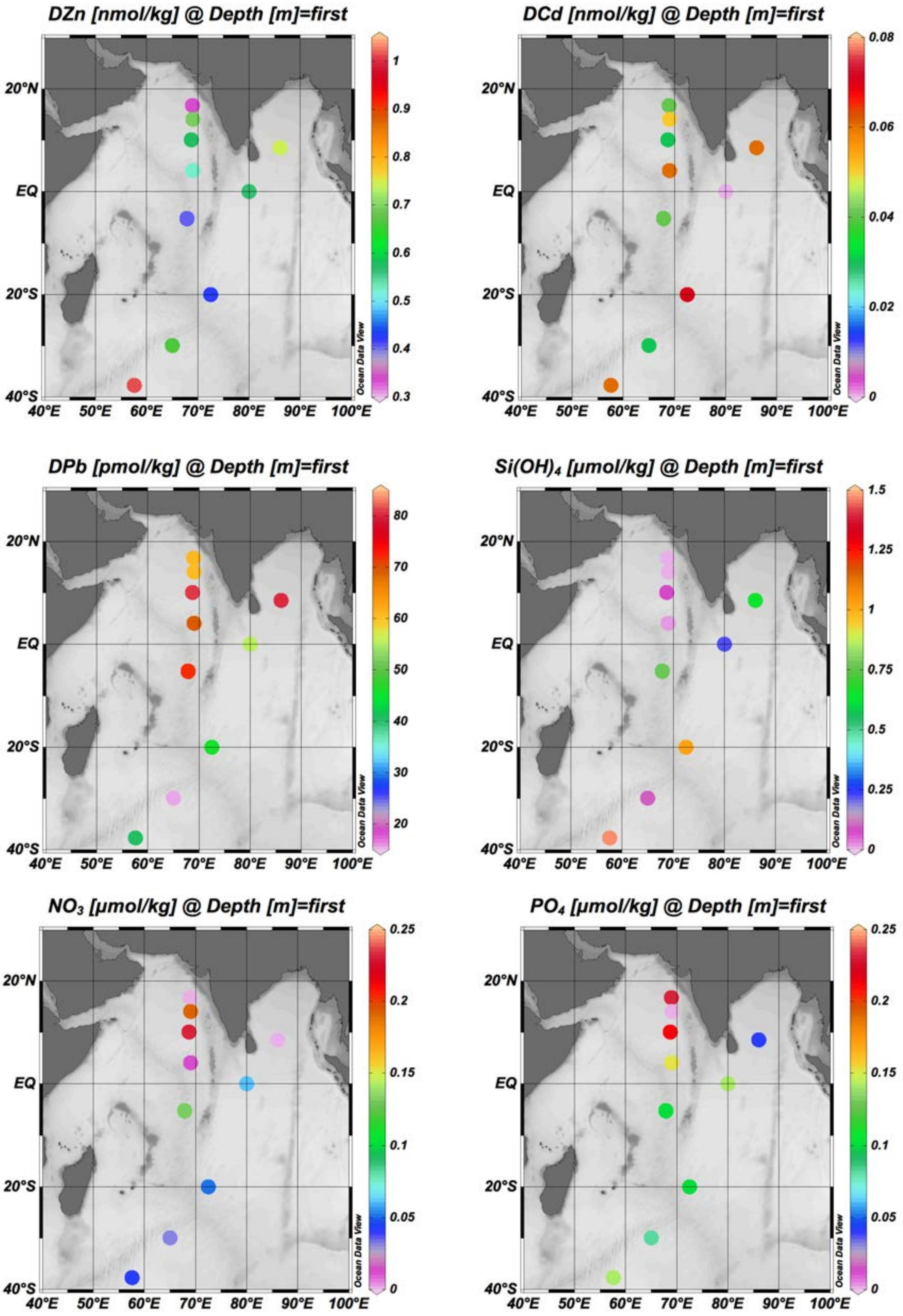

Supplementary Figure 5 b. Surface distribution of DZn, DCd, DPb, silicate, nitrate, and phosphate.

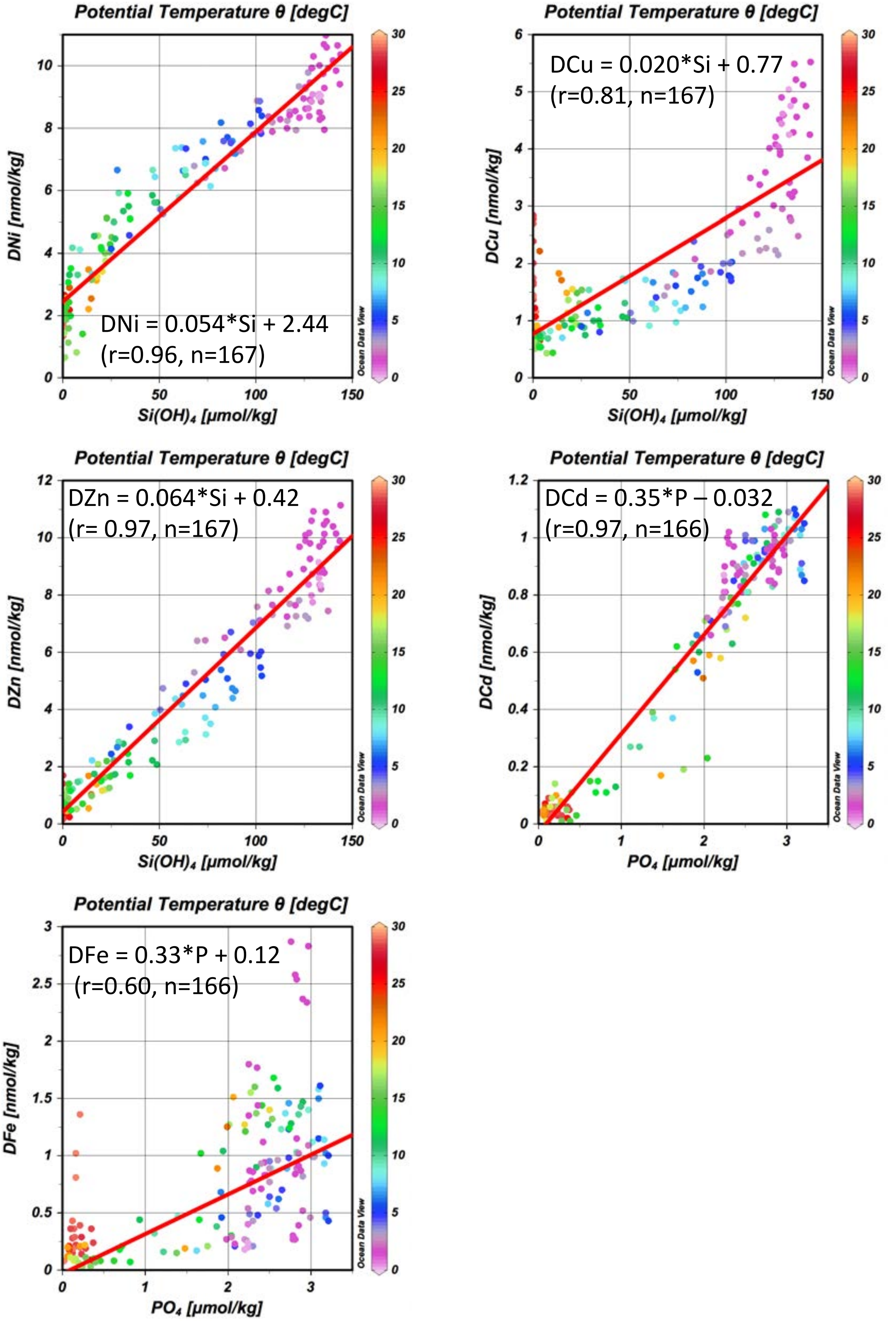

Supplementary Figure 6. Plots of DMs vs. nutrients. The p value for each regression line is less than 0.01. See Supplementary Table 2 for comparison with literature data.

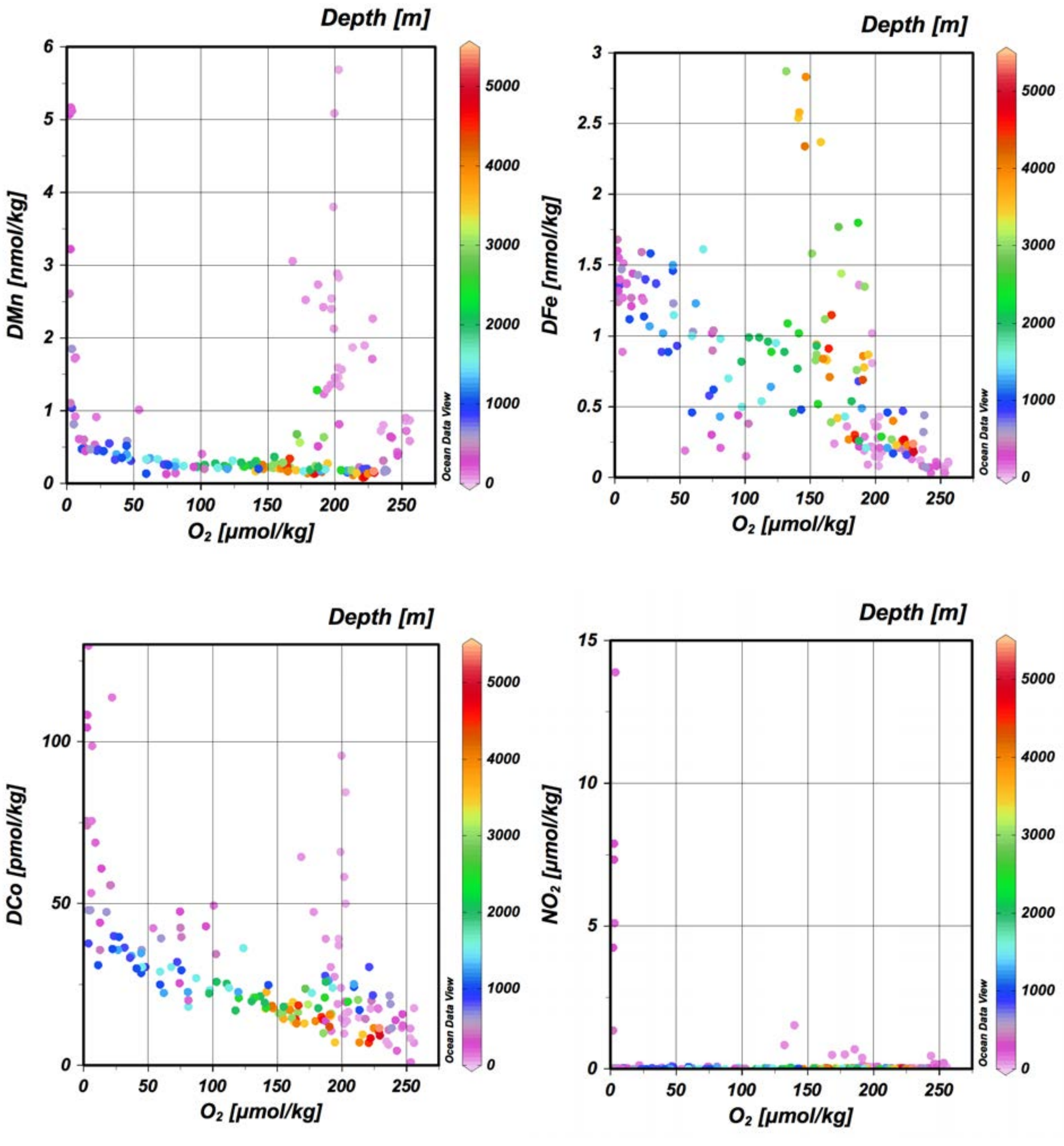

Supplementary Figure 7. Plots of DMs and nitrite vs. oxygen. In the oxygen minimum zone (OMZ), DMn, DCo, and nitrite increased below a certain threshold oxygen concentration: ~20 µmol/kg for DCo, ~10 µmol/kg for DMn, and ~4 µmol/kg for nitrite. At the surface, DMn increased ~29-fold compared to the background concentration, with oxygen concentration of ~200 µmol/kg, indicating photochemical reduction. The increase in DFe and DCo was not as high as that of DMn due to biological uptake.

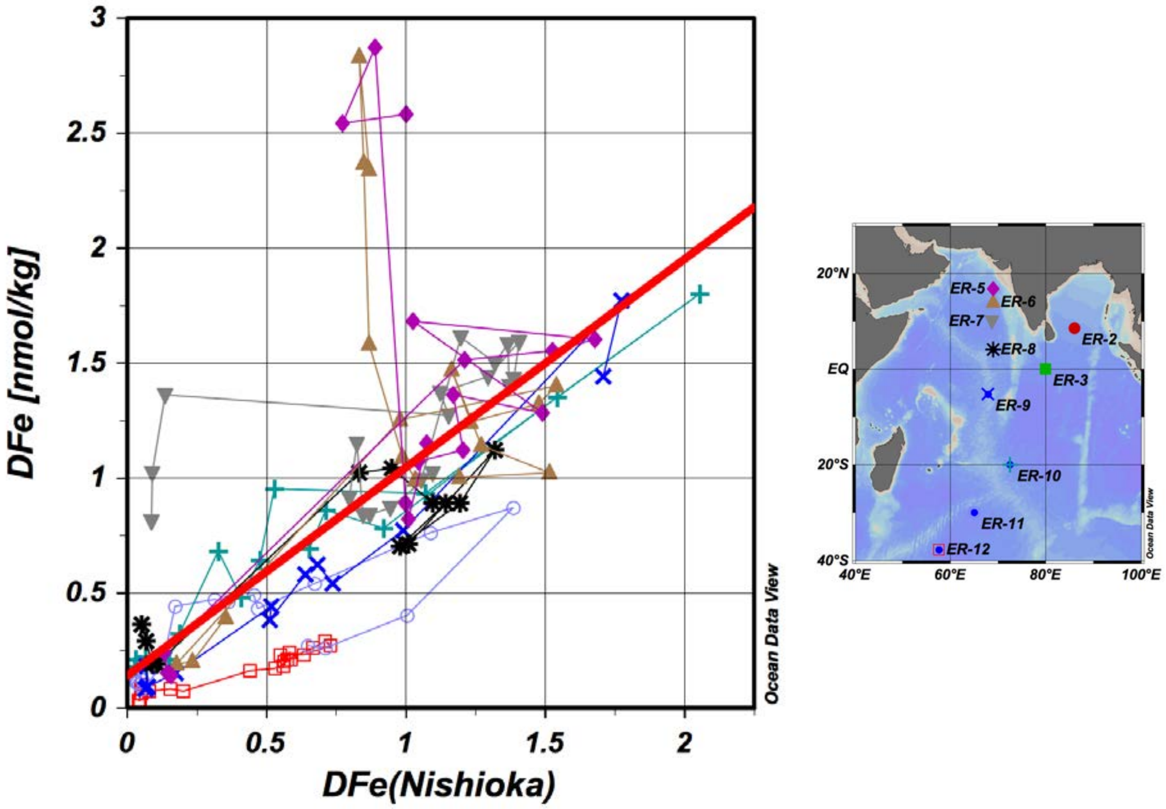

Supplementary Figure 8. Plots of DFe in this study vs. DFe in Nishioka et al. 2013. The regression line for all data is

DFe [nmol/kg] = 0.91*DFe(Nishioka) [nmol/L] + 0.14 (r = 0.71, n = 132, $p < 0.01$)

showing a significant positive intercept. The largest differences are observed for deep water samples at ER5 and ER6. These discrepancies are likely caused by that a larger portion of dissolved pool was determined in this study due to ~1.5 y long storage of acidified samples.
